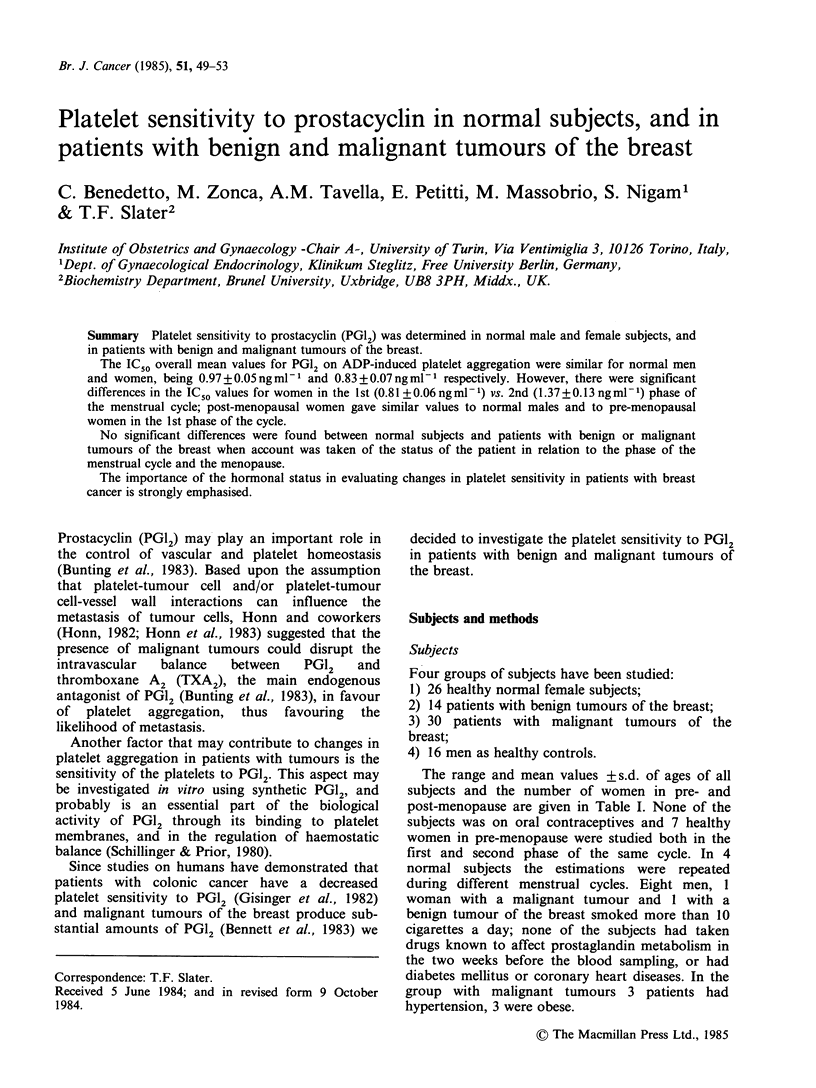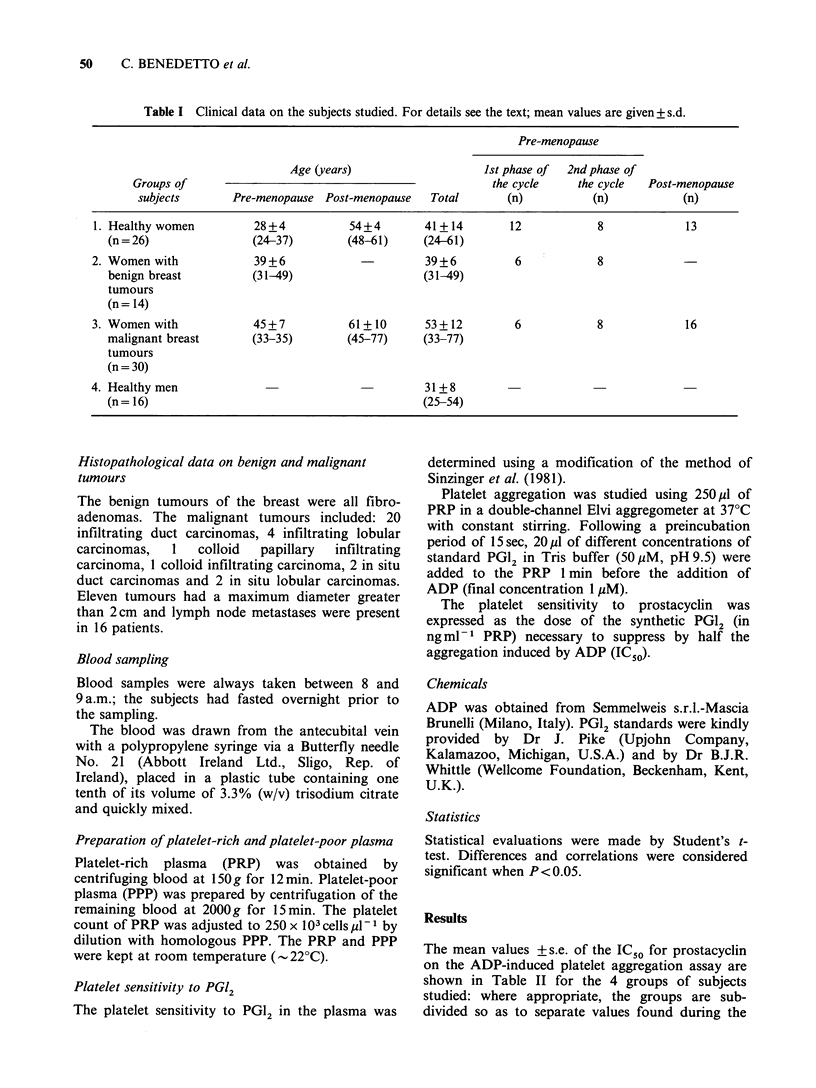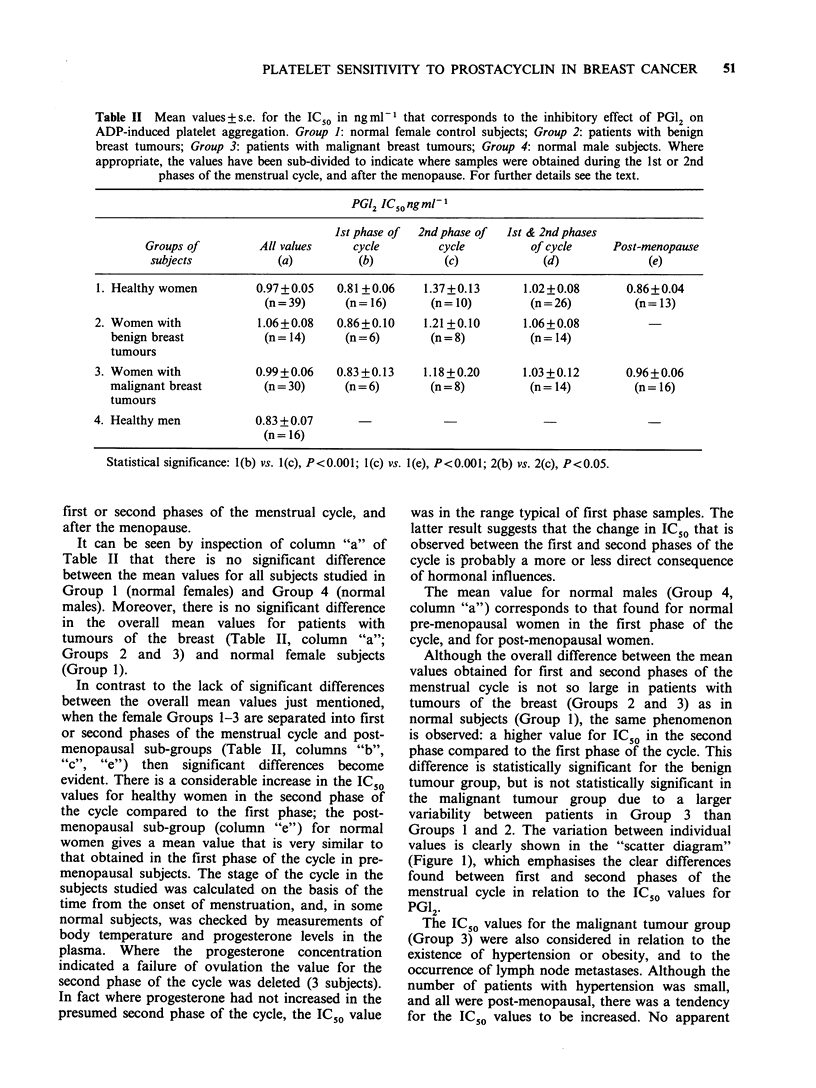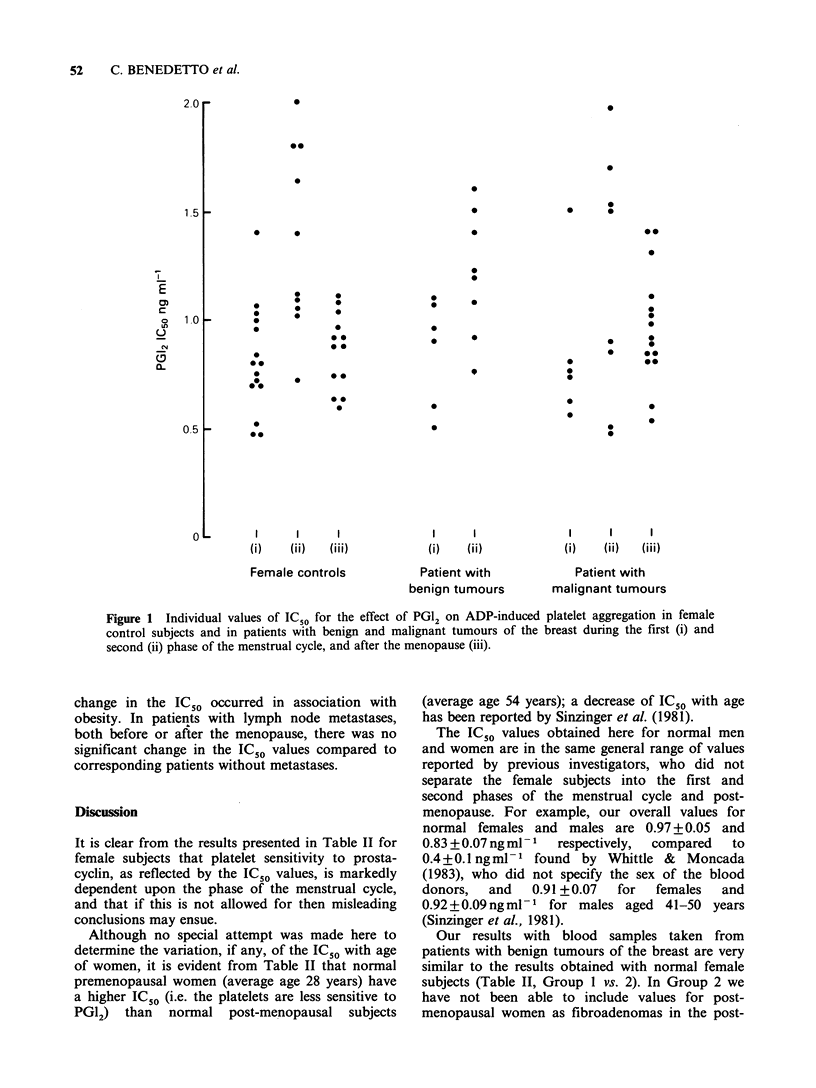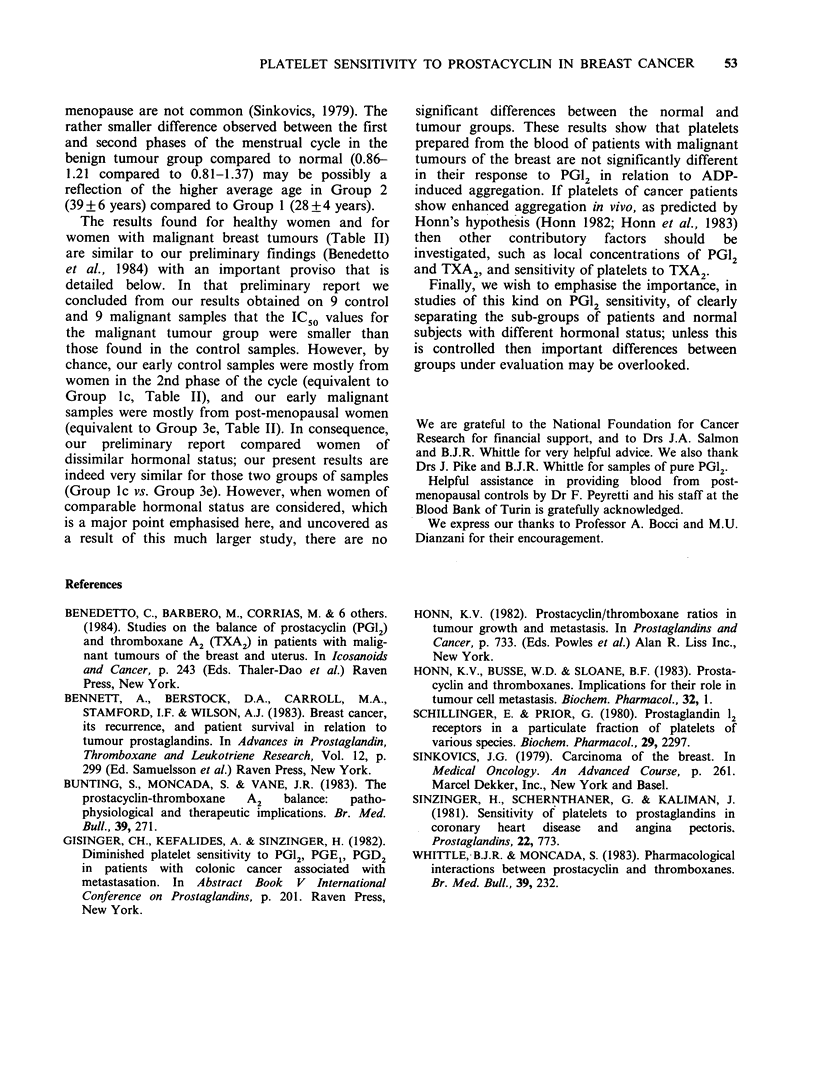# Platelet sensitivity to prostacyclin in normal subjects, and in patients with benign and malignant tumours of the breast.

**DOI:** 10.1038/bjc.1985.7

**Published:** 1985-01

**Authors:** C. Benedetto, M. Zonca, A. M. Tavella, E. Petitti, M. Massobrio, S. Nigam, T. F. Slater

## Abstract

Platelet sensitivity to prostacyclin (PG12) was determined in normal male and female subjects, and in patients with benign and malignant tumours of the breast. The IC50 overall mean values for PG12 on ADP-induced platelet aggregation were similar for normal men and women, being 0.97 +/- 0.05 ng ml-1 and 0.83 +/- 0.07 ng ml-1 respectively. However, there were significant differences in the IC50 values for women in the 1st (0.81 +/- 0.06 ng ml-1) vs. 2nd (1.37 +/- 0.13 ng ml-1) phase of the menstrual cycle; post-menopausal women gave similar values to normal males and to pre-menopausal women in the 1st phase of the cycle. No significant differences were found between normal subjects and patients with benign or malignant tumours of the breast when account was taken of the status of the patient in relation to the phase of the menstrual cycle and the menopause. The importance of the hormonal status in evaluating changes in platelet sensitivity in patients with breast cancer is strongly emphasised.


Br. J. Cancer (1985), 51, 49–53

### Platelet sensitivity to prostacyclin in normal subjects, and in patients with benign and malignant tumours of the breast

C. Benedetto, M. Zonca, A.M. Tavella, E. Petitti, M. Massobrio, S. Nigam[1] & T.F. Slater[2]

*Institute of Obstetrics and Gynaecology -Chair A-, University of Turin, Via Ventimiglia 3, 10126 Torino, Italy,*
*[1]Dept. of Gynaecological Endocrinology, Klinikum Steglitz, Free University Berlin, Germany,*
*[2]Biochemistry Department, Brunel University, Uxbridge, UB8 3PH, Middx., UK.*

**Summary** Platelet sensitivity to prostacyclin ($PGI_2$) was determined in normal male and female subjects, and in patients with benign and malignant tumours of the breast.

The $IC_{50}$ overall mean values for $PGI_2$ on ADP-induced platelet aggregation were similar for normal men and women, being $0.97 \pm 0.05\,ng\,ml^{-1}$ and $0.83 \pm 0.07\,ng\,ml^{-1}$ respectively. However, there were significant differences in the $IC_{50}$ values for women in the 1st ($0.81 \pm 0.06\,ng\,ml^{-1}$) *vs.* 2nd ($1.37 \pm 0.13\,ng\,ml^{-1}$) phase of the menstrual cycle; post-menopausal women gave similar values to normal males and to pre-menopausal women in the 1st phase of the cycle.

No significant differences were found between normal subjects and patients with benign or malignant tumours of the breast when account was taken of the status of the patient in relation to the phase of the menstrual cycle and the menopause.

The importance of the hormonal status in evaluating changes in platelet sensitivity in patients with breast cancer is strongly emphasised.

Prostacyclin ($PGI_2$) may play an important role in the control of vascular and platelet homeostasis (Bunting *et al.*, 1983). Based upon the assumption that platelet-tumour cell and/or platelet-tumour cell-vessel wall interactions can influence the metastasis of tumour cells, Honn and coworkers (Honn, 1982; Honn *et al.*, 1983) suggested that the presence of malignant tumours could disrupt the intravascular balance between $PGI_2$ and thromboxane $A_2$ ($TXA_2$), the main endogenous antagonist of $PGI_2$ (Bunting *et al.*, 1983), in favour of platelet aggregation, thus favouring the likelihood of metastasis.

Another factor that may contribute to changes in platelet aggregation in patients with tumours is the sensitivity of the platelets to $PGI_2$. This aspect may be investigated *in vitro* using synthetic $PGI_2$, and probably is an essential part of the biological activity of $PGI_2$ through its binding to platelet membranes, and in the regulation of haemostatic balance (Schillinger & Prior, 1980).

Since studies on humans have demonstrated that patients with colonic cancer have a decreased platelet sensitivity to $PGI_2$ (Gisinger *et al.*, 1982) and malignant tumours of the breast produce substantial amounts of $PGI_2$ (Bennett *et al.*, 1983) we decided to investigate the platelet sensitivity to $PGI_2$ in patients with benign and malignant tumours of the breast.

Correspondence: T.F. Slater.
Received 5 June 1984; and in revised form 9 October 1984.

### Subjects and methods

### *Subjects*

Four groups of subjects have been studied:
1) 26 healthy normal female subjects;
2) 14 patients with benign tumours of the breast;
3) 30 patients with malignant tumours of the breast;
4) 16 men as healthy controls.

The range and mean values ±s.d. of ages of all subjects and the number of women in pre- and post-menopause are given in Table I. None of the subjects was on oral contraceptives and 7 healthy women in pre-menopause were studied both in the first and second phase of the same cycle. In 4 normal subjects the estimations were repeated during different menstrual cycles. Eight men, 1 woman with a malignant tumour and 1 with a benign tumour of the breast smoked more than 10 cigarettes a day; none of the subjects had taken drugs known to affect prostaglandin metabolism in the two weeks before the blood sampling, or had diabetes mellitus or coronary heart diseases. In the group with malignant tumours 3 patients had hypertension, 3 were obese.

© The Macmillan Press Ltd., 1985

**Table I** Clinical data on the subjects studied. For details see the text; mean values are given ± s.d.

| Groups of subjects | Age (years) | | | Pre-menopause | | |
|---|---|---|---|---|---|---|
| | Pre-menopause | Post-menopause | Total | 1st phase of the cycle (n) | 2nd phase of the cycle (n) | Post-menopause (n) |
| 1. Healthy women (n = 26) | 28 ± 4 (24–37) | 54 ± 4 (48–61) | 41 ± 14 (24–61) | 12 | 8 | 13 |
| 2. Women with benign breast tumours (n = 14) | 39 ± 6 (31–49) | — | 39 ± 6 (31–49) | 6 | 8 | — |
| 3. Women with malignant breast tumours (n = 30) | 45 ± 7 (33–35) | 61 ± 10 (45–77) | 53 ± 12 (33–77) | 6 | 8 | 16 |
| 4. Healthy men (n = 16) | — | — | 31 ± 8 (25–54) | — | — | — |

### *Histopathological data on benign and malignant tumours*

The benign tumours of the breast were all fibroadenomas. The malignant tumours included: 20 infiltrating duct carcinomas, 4 infiltrating lobular carcinomas, 1 colloid papillary infiltrating carcinoma, 1 colloid infiltrating carcinoma, 2 in situ duct carcinomas and 2 in situ lobular carcinomas. Eleven tumours had a maximum diameter greater than 2 cm and lymph node metastases were present in 16 patients.

### *Blood sampling*

Blood samples were always taken between 8 and 9 a.m.; the subjects had fasted overnight prior to the sampling.

The blood was drawn from the antecubital vein with a polypropylene syringe via a Butterfly needle No. 21 (Abbott Ireland Ltd., Sligo, Rep. of Ireland), placed in a plastic tube containing one tenth of its volume of 3.3% (w/v) trisodium citrate and quickly mixed.

### *Preparation of platelet-rich and platelet-poor plasma*

Platelet-rich plasma (PRP) was obtained by centrifuging blood at 150 *g* for 12 min. Platelet-poor plasma (PPP) was prepared by centrifugation of the remaining blood at 2000 *g* for 15 min. The platelet count of PRP was adjusted to $250 \times 10^3$ cells $\mu l^{-1}$ by dilution with homologous PPP. The PRP and PPP were kept at room temperature (~22°C).

### *Platelet sensitivity to $PGI_2$*

The platelet sensitivity to $PGI_2$ in the plasma was determined using a modification of the method of Sinzinger *et al.* (1981).

Platelet aggregation was studied using 250 $\mu$l of PRP in a double-channel Elvi aggregometer at 37°C with constant stirring. Following a preincubation period of 15 sec, 20 $\mu$l of different concentrations of standard $PGI_2$ in Tris buffer (50 $\mu$M, pH 9.5) were added to the PRP 1 min before the addition of ADP (final concentration 1 $\mu$M).

The platelet sensitivity to prostacyclin was expressed as the dose of the synthetic $PGI_2$ (in ng $ml^{-1}$ PRP) necessary to suppress by half the aggregation induced by ADP ($IC_{50}$).

### *Chemicals*

ADP was obtained from Semmelweis s.r.l.-Mascia Brunelli (Milano, Italy). $PGI_2$ standards were kindly provided by Dr J. Pike (Upjohn Company, Kalamazoo, Michigan, U.S.A.) and by Dr B.J.R. Whittle (Wellcome Foundation, Beckenham, Kent, U.K.).

### *Statistics*

Statistical evaluations were made by Student's *t*-test. Differences and correlations were considered significant when $P < 0.05$.

### Results

The mean values ± s.e. of the $IC_{50}$ for prostacyclin on the ADP-induced platelet aggregation assay are shown in Table II for the 4 groups of subjects studied: where appropriate, the groups are subdivided so as to separate values found during the

**Table II** Mean values ± s.e. for the $IC_{50}$ in $ng\,ml^{-1}$ that corresponds to the inhibitory effect of $PGI_2$ on ADP-induced platelet aggregation. *Group 1*: normal female control subjects; *Group 2*: patients with benign breast tumours; *Group 3*: patients with malignant breast tumours; *Group 4*: normal male subjects. Where appropriate, the values have been sub-divided to indicate where samples were obtained during the 1st or 2nd phases of the menstrual cycle, and after the menopause. For further details see the text.

| | *$PGI_2$ $IC_{50}$ $ng\,ml^{-1}$* | | | | |
|---|---|---|---|---|---|
| *Groups of subjects* | *All values* (*a*) | *1st phase of cycle* (*b*) | *2nd phase of cycle* (*c*) | *1st & 2nd phases of cycle* (*d*) | *Post-menopause* (*e*) |
| 1. Healthy women | 0.97±0.05 (n=39) | 0.81±0.06 (n=16) | 1.37±0.13 (n=10) | 1.02±0.08 (n=26) | 0.86±0.04 (n=13) |
| 2. Women with benign breast tumours | 1.06±0.08 (n=14) | 0.86±0.10 (n=6) | 1.21±0.10 (n=8) | 1.06±0.08 (n=14) | — |
| 3. Women with malignant breast tumours | 0.99±0.06 (n=30) | 0.83±0.13 (n=6) | 1.18±0.20 (n=8) | 1.03±0.12 (n=14) | 0.96±0.06 (n=16) |
| 4. Healthy men | 0.83±0.07 (n=16) | — | — | — | — |

Statistical significance: 1(b) *vs.* 1(c), $P<0.001$; 1(c) *vs.* 1(e), $P<0.001$; 2(b) *vs.* 2(c), $P<0.05$.

first or second phases of the menstrual cycle, and after the menopause.

It can be seen by inspection of column "a" of Table II that there is no significant difference between the mean values for all subjects studied in Group 1 (normal females) and Group 4 (normal males). Moreover, there is no significant difference in the overall mean values for patients with tumours of the breast (Table II, column "a"; Groups 2 and 3) and normal female subjects (Group 1).

In contrast to the lack of significant differences between the overall mean values just mentioned, when the female Groups 1–3 are separated into first or second phases of the menstrual cycle and post-menopausal sub-groups (Table II, columns "b", "c", "e") then significant differences become evident. There is a considerable increase in the $IC_{50}$ values for healthy women in the second phase of the cycle compared to the first phase; the post-menopausal sub-group (column "e") for normal women gives a mean value that is very similar to that obtained in the first phase of the cycle in pre-menopausal subjects. The stage of the cycle in the subjects studied was calculated on the basis of the time from the onset of menstruation, and, in some normal subjects, was checked by measurements of body temperature and progesterone levels in the plasma. Where the progesterone concentration indicated a failure of ovulation the value for the second phase of the cycle was deleted (3 subjects). In fact where progesterone had not increased in the presumed second phase of the cycle, the $IC_{50}$ value was in the range typical of first phase samples. The latter result suggests that the change in $IC_{50}$ that is observed between the first and second phases of the cycle is probably a more or less direct consequence of hormonal influences.

The mean value for normal males (Group 4, column "a") corresponds to that found for normal pre-menopausal women in the first phase of the cycle, and for post-menopausal women.

Although the overall difference between the mean values obtained for first and second phases of the menstrual cycle is not so large in patients with tumours of the breast (Groups 2 and 3) as in normal subjects (Group 1), the same phenomenon is observed: a higher value for $IC_{50}$ in the second phase compared to the first phase of the cycle. This difference is statistically significant for the benign tumour group, but is not statistically significant in the malignant tumour group due to a larger variability between patients in Group 3 than Groups 1 and 2. The variation between individual values is clearly shown in the "scatter diagram" (Figure 1), which emphasises the clear differences found between first and second phases of the menstrual cycle in relation to the $IC_{50}$ values for $PGI_2$.

The $IC_{50}$ values for the malignant tumour group (Group 3) were also considered in relation to the existence of hypertension or obesity, and to the occurrence of lymph node metastases. Although the number of patients with hypertension was small, and all were post-menopausal, there was a tendency for the $IC_{50}$ values to be increased. No apparent

**Figure 1** Individual values of $IC_{50}$ for the effect of $PGI_2$ on ADP-induced platelet aggregation in female control subjects and in patients with benign and malignant tumours of the breast during the first (i) and second (ii) phase of the menstrual cycle, and after the menopause (iii).

change in the $IC_{50}$ occurred in association with obesity. In patients with lymph node metastases, both before or after the menopause, there was no significant change in the $IC_{50}$ values compared to corresponding patients without metastases.

### Discussion

It is clear from the results presented in Table II for female subjects that platelet sensitivity to prostacyclin, as reflected by the $IC_{50}$ values, is markedly dependent upon the phase of the menstrual cycle, and that if this is not allowed for then misleading conclusions may ensue.

Although no special attempt was made here to determine the variation, if any, of the $IC_{50}$ with age of women, it is evident from Table II that normal premenopausal women (average age 28 years) have a higher $IC_{50}$ (i.e. the platelets are less sensitive to $PGI_2$) than normal post-menopausal subjects (average age 54 years); a decrease of $IC_{50}$ with age has been reported by Sinzinger *et al.* (1981).

The $IC_{50}$ values obtained here for normal men and women are in the same general range of values reported by previous investigators, who did not separate the female subjects into the first and second phases of the menstrual cycle and post-menopause. For example, our overall values for normal females and males are $0.97 \pm 0.05$ and $0.83 \pm 0.07\ \text{ng ml}^{-1}$ respectively, compared to $0.4 \pm 0.1\ \text{ng ml}^{-1}$ found by Whittle & Moncada (1983), who did not specify the sex of the blood donors, and $0.91 \pm 0.07$ for females and $0.92 \pm 0.09\ \text{ng ml}^{-1}$ for males aged 41–50 years (Sinzinger *et al.*, 1981).

Our results with blood samples taken from patients with benign tumours of the breast are very similar to the results obtained with normal female subjects (Table II, Group 1 *vs.* 2). In Group 2 we have not been able to include values for post-menopausal women as fibroadenomas in the postmenopause are not common (Sinkovics, 1979). The rather smaller difference observed between the first and second phases of the menstrual cycle in the benign tumour group compared to normal (0.86–1.21 compared to 0.81–1.37) may be possibly a reflection of the higher average age in Group 2 ($39 \pm 6$ years) compared to Group 1 ($28 \pm 4$ years).

The results found for healthy women and for women with malignant breast tumours (Table II) are similar to our preliminary findings (Benedetto *et al.*, 1984) with an important proviso that is detailed below. In that preliminary report we concluded from our results obtained on 9 control and 9 malignant samples that the $IC_{50}$ values for the malignant tumour group were smaller than those found in the control samples. However, by chance, our early control samples were mostly from women in the 2nd phase of the cycle (equivalent to Group 1c, Table II), and our early malignant samples were mostly from post-menopausal women (equivalent to Group 3e, Table II). In consequence, our preliminary report compared women of dissimilar hormonal status; our present results are indeed very similar for those two groups of samples (Group 1c *vs.* Group 3e). However, when women of comparable hormonal status are considered, which is a major point emphasised here, and uncovered as a result of this much larger study, there are no significant differences between the normal and tumour groups. These results show that platelets prepared from the blood of patients with malignant tumours of the breast are not significantly different in their response to $PGI_2$ in relation to ADP-induced aggregation. If platelets of cancer patients show enhanced aggregation *in vivo*, as predicted by Honn's hypothesis (Honn 1982; Honn *et al.*, 1983) then other contributory factors should be investigated, such as local concentrations of $PGI_2$ and $TXA_2$, and sensitivity of platelets to $TXA_2$.

Finally, we wish to emphasise the importance, in studies of this kind on $PGI_2$ sensitivity, of clearly separating the sub-groups of patients and normal subjects with different hormonal status; unless this is controlled then important differences between groups under evaluation may be overlooked.

We are grateful to the National Foundation for Cancer Research for financial support, and to Drs J.A. Salmon and B.J.R. Whittle for very helpful advice. We also thank Drs J. Pike and B.J.R. Whittle for samples of pure $PGI_2$.

Helpful assistance in providing blood from post-menopausal controls by Dr F. Peyretti and his staff at the Blood Bank of Turin is gratefully acknowledged.

We express our thanks to Professor A. Bocci and M.U. Dianzani for their encouragement.

### References

BENEDETTO, C., BARBERO, M., CORRIAS, M. & 6 others. (1984). Studies on the balance of prostacyclin ($PGI_2$) and thromboxane $A_2$ ($TXA_2$) in patients with malignant tumours of the breast and uterus. In *Icosanoids and Cancer*, p. 243 (Eds. Thaler-Dao *et al.*) Raven Press, New York.

BENNETT, A., BERSTOCK, D.A., CARROLL, M.A., STAMFORD, I.F. & WILSON, A.J. (1983). Breast cancer, its recurrence, and patient survival in relation to tumour prostaglandins. In *Advances in Prostaglandin, Thromboxane and Leukotriene Research*, Vol. 12, p. 299 (Ed. Samuelsson *et al.*) Raven Press, New York.

BUNTING, S., MONCADA, S. & VANE, J.R. (1983). The prostacyclin-thromboxane $A_2$ balance: pathophysiological and therapeutic implications. *Br. Med. Bull.*, **39**, 271.

GISINGER, CH., KEFALIDES, A. & SINZINGER, H. (1982). Diminished platelet sensitivity to $PGI_2$, $PGE_1$, $PGD_2$ in patients with colonic cancer associated with metastasation. In *Abstract Book V International Conference on Prostaglandins*, p. 201. Raven Press, New York.

HONN, K.V. (1982). Prostacyclin/thromboxane ratios in tumour growth and metastasis. In *Prostaglandins and Cancer*, p. 733. (Eds. Powles *et al.*) Alan R. Liss Inc., New York.

HONN, K.V., BUSSE, W.D. & SLOANE, B.F. (1983). Prostacyclin and thromboxanes. Implications for their role in tumour cell metastasis. *Biochem. Pharmacol.*, **32**, 1.

SCHILLINGER, E. & PRIOR, G. (1980). Prostaglandin $I_2$ receptors in a particulate fraction of platelets of various species. *Biochem. Pharmacol.*, **29**, 2297.

SINKOVICS, J.G. (1979). Carcinoma of the breast. In *Medical Oncology. An Advanced Course*, p. 261. Marcel Dekker, Inc., New York and Basel.

SINZINGER, H., SCHERNTHANER, G. & KALIMAN, J. (1981). Sensitivity of platelets to prostaglandins in coronary heart disease and angina pectoris. *Prostaglandins*, **22**, 773.

WHITTLE, B.J.R. & MONCADA, S. (1983). Pharmacological interactions between prostacyclin and thromboxanes. *Br. Med. Bull.*, **39**, 232.